# Barriers and Enablers to Optimal Antimicrobial Use in Respiratory Tract Infections

**DOI:** 10.3390/antibiotics14101039

**Published:** 2025-10-16

**Authors:** Savannah Reali, Jin-Gun Cho, Jan-Willem Alffenaar, Parisa Aslani

**Affiliations:** 1 School of Pharmacy, Faculty of Medicine and Health, The University of Sydney, Sydney, NSW 2050, Australia; savannah.reali@sydney.edu.au (S.R.);; 2 Westmead Hospital, Sydney, NSW 2145, Australia; 3 Sydney Infectious Diseases Institute (Sydney ID), The University of Sydney, Sydney, NSW 2145, Australia; 4 Department of Respiratory and Sleep Medicine, Westmead Hospital, Sydney, NSW 2145, Australia; 5 Sydney Medical School, Faculty of Medicine and Health, The University of Sydney, Sydney, NSW 2050, Australia

**Keywords:** antimicrobial stewardship, prescribing guidelines, optimisation, respiratory tract infection, clinical practice

## Abstract

**Background/Objectives**: Antibiotic use for respiratory tract infections is often suboptimal and does not follow clinical guidelines. Inappropriate antibiotic use is a driver for antimicrobial resistance. Our aims were to identify antimicrobial prescribing guidelines used to aid decision-making, describe perceptions on guideline compliance, and explore barriers and enablers to optimal antimicrobial use in hospitalised patients with respiratory tract infections. **Methods**: Qualitative semi-structured interviews were conducted with antimicrobial stewardship pharmacists (*n* = 10) and respiratory (*n* = 5) and infectious diseases (*n* = 2) specialists from hospitals in New South Wales, Australia. Interviews were recorded, transcribed verbatim, and coded thematically. **Results**: Participants primarily used an online evidence-based national consensus guideline to inform antibiotic choices. These guidelines were perceived to be useful and simple to access but did not contain all relevant information and were deemed too verbose to be practical. Pharmacists and physicians had different perceptions on guideline compliance. Barriers to guideline compliance and optimal antibiotic use included inadequate diagnostics and staffing, patient treatment pressure, and lack of knowledge and ownership of the impact of prescribing decisions. A positive working relationship with the antimicrobial stewardship team, prescribing feedback, and increasing the availability of data and decision support tools were reported as enablers. **Conclusions**: National prescribing guidelines are available to guide decisions but adherence to their recommendations was variable. Insufficient access to useful diagnostics, resourcing, and knowledge may negatively impact antimicrobial prescribing. Education, feedback, and tools and data to aid decision-making may improve optimal antimicrobial use. Focusing on incorporating these enablers into future prescribing interventions will be vital for antimicrobial stewardship practices.

## 1. Introduction

Respiratory tract infections (RTIs) are one of the most common indications for all antimicrobial prescriptions in the hospital setting and are amongst the most challenging diseases for clinical management [1,2,3]. They have some of the highest rates of antimicrobial prescribing guideline non-compliance, in particular for pneumonia and exacerbations of chronic obstructive pulmonary disease [1]. Overuse and inappropriate use of antimicrobials contributes to the problem of antimicrobial resistance (AMR). The World Health Organization’s Global Action Plan on AMR calls for an urgent global effort to limit the devastating effects of AMR [4].

Many interventions aimed at improving antibiotic prescribing practices have been developed and tested. A 2017 Cochrane Review found enablement and restriction interventions increased prescribing compliance with policies and reduced duration of antibiotic therapy and length of hospital stay without increased adverse patient outcomes [5]. Focusing on RTIs specifically, a 2018 systematic review found patient and clinician education, procalcitonin testing, and electronic decision support systems all improved antibiotic prescribing [6]. Despite the published success of interventions aimed at improving prescribing, the overall appropriateness of antimicrobial use has remained consistently suboptimal [1]. Half of antimicrobials prescribed in hospitals in Bhutan [7], one-fifth in Canada [8], and one-quarter in Australia [1] are inappropriate. It is evident that increased knowledge and awareness of AMR and implementation of antimicrobial stewardship (AMS) programs alone is not sufficient to address this. There are clearly barriers preventing physicians from making optimal antimicrobial prescribing choices, and further investigation into these barriers and how they can be addressed is warranted.

Previous studies have identified several barriers and enablers to optimal antimicrobial prescribing in RTIs. Barriers previously reported in primary care include patient pressure to prescribe, persuasive information from drug representatives, lack of knowledge on the pathogenesis of RTIs, and lack of access to updated resistance data and antibiotic guidelines that discuss the complexities of real-life patients. Feedback on prescribing habits, penalties for inappropriate prescribing, and training on ways to communicate with patients about AMR were reported as enablers [9,10]. Barriers reported from hospital physicians include scepticism of applicability of guideline recommendations to their patients, lack of concern about the threat of AMR, medical hierarchy limiting junior physicians’ decision-making capacity, complacency in accessing guidelines, lack of feedback on prescribing, and difficulty diagnosing and distinguishing between types of RTIs, their pathogenesis, and distinguishing between colonisation or infection. Enablers included knowledge and positive attitude towards prescribing guidelines, audits, and educational interventions [11,12]. This study will add to this body of literature by providing a more in-depth discussion of views on prescribing guidelines and comparing barriers and enablers between different hospital settings.

The aims of this study are therefore to identify antimicrobial prescribing guidelines used to aid decision-making, describe perceptions on guideline compliance, and explore barriers and enablers to optimal antimicrobial use in hospitalised patients with RTIs.

## 2. Results

Ten AMS pharmacists, two infectious diseases staff specialists, and five respiratory staff specialists were interviewed (seventeen participants in total). Ten were female and seven were male. One participant worked in a private hospital as their primary place of practice. Sixteen worked in a public hospital as their primary place of practice, and three of these also worked in a private hospital. Twelve participants worked in a metropolitan facility and five worked in a regional facility. Five participants had 0–3 years of experience as a specialist, nine had 4–9 years’ experience, and three had 10 or more years’ experience. The average interview length was 39 min (ranging from 27 min to 48 min).

The roles of interviewed AMS pharmacists within their hospitals included AMS rounds (post-prescription review of antimicrobials prescribed in the hospital), antimicrobial audits, education, antimicrobial projects, attendance and participation in AMS committee meetings, and reviewing antimicrobial policies and procedures. Whilst AMS pharmacists do not have authority to prescribe antimicrobials in Australia, they often collaborate with physicians and provide advice and recommendations to optimise antimicrobial therapy. The roles of interviewed staff specialists within their hospitals included admitting patients, diagnosis, instigating treatment (including prescribing antimicrobials), ongoing review and management of patients, taking consults, teaching and supervising junior staff, and being on-call. Infectious diseases staff specialists additionally mentioned participation in AMS rounds and AMS committee meetings.

There were five main themes extrapolated from the data: resources used to guide antimicrobial decisions, healthcare professionals’ opinions of those resources, perceived compliance with prescribing guidelines, barriers to optimal prescribing, and enablers for optimal prescribing (Table 1). Data is reported from both AMS pharmacists and physicians together, unless otherwise specified. More participant quotes can be found in Supplement S1.

### 2.1. Resources Used to Guide Antimicrobial Decisions

Sixteen of the seventeen participants reported using the Therapeutic Guidelines, an online evidence-based Australian consensus guideline, to inform their antimicrobial decisions [13].

*“Mainly the eTG [electronic Therapeutic Guidelines] guideline… they’ve presumably looked at the Australian data and they’re smarter than me, they know more than me, so I may as well just follow what they say.”*

—P11, Respiratory Staff Specialist.

Only one participant did not use the Therapeutic Guidelines as they found their recommendations limited.

Some hospitals had local policies and procedures for the management of RTIs, in particular community-acquired pneumonia (CAP) and coronavirus disease (COVID-19). COVID-19 management was widely primarily institution-specific.

Some institutions developed their own CAP guidelines with specific intentions to alter regimens set out in the Therapeutic Guidelines due to local population differences compared to the national standard or to provide more rationale for the choices. These were primarily developed and used by AMS pharmacists.

*“So, generally the population in [local health district] are… considered to be at high risk of multi-drug-resistant organisms… So, I guess the acuity of the district drove the need to have a local guideline which might recommend more broader antibiotics.”*

—P3, AMS Pharmacist.

Participants reported using other guidelines when the Therapeutic Guidelines did not have sufficient information to guide antimicrobial treatment. This was particularly relevant for complex patients or those with rare pathogens. These other resources included primary literature, the Thoracic Society of Australia and New Zealand, Australian Medicines Handbook and Renal Drug Handbook for drug information, the Australasian Consensus Guidelines for the use of antifungal agents, and international guidelines including UpToDate [14], the WHO for tuberculosis therapy, The Sanford Guide to Antimicrobial Therapy, and the European Respiratory Society.

*“And it would be a rare occasion that I have to like consult, you know, the literature or something. Maybe it’s like I found a weird organism or something like that.”*

—P10, Respiratory Staff Specialist.

*“So that’s why I’m using UpToDate. For the more, I guess, complex patient or where the information is lacking in the Therapeutic Guidelines.”*

—P4, AMS Pharmacist.

Finally, physicians often reported consulting with AMS or infectious diseases specialists for complex patients, those infected with unusual pathogens, or for those with severe infections in the Intensive Care Unit.

*“Yes, if patients definitely got complicated infection, then we always have an Infectious Disease consult or speak to AMS for approval of antibiotics.”*

—P7, Respiratory Staff Specialist.

### 2.2. Healthcare Professionals’ Opinions on Antimicrobial Guidelines

Participants discussed the applicability of the recommendations in prescribing guidelines to their patients. Prescribing guidelines refer to the Australian Therapeutic Guidelines unless otherwise specified [13]. They felt that recommendations in the Therapeutic Guidelines were appropriate and applicable as they were based on local data.

*“Applicability in like most cases, it’s a pretty good match.”*

—P8, AMS Pharmacist.

There were some specific situations in which participants did not agree with the recommendations in the guidelines.

*“I’m not the biggest fan of their suggestion of intravenous azithromycin in severe pneumonia, because I don’t think anyone ever needs intravenous azithromycin unless they’re completely nil by mouth.”*

—P13, AMS Pharmacist.

Participants also discussed issues implementing recommendations from international guidelines as they are not always transferable to the local context due to differences in patterns of resistance and antimicrobial availability.

*“Because you do notice some differences in the availability of the drugs.... Some of them are applicable but some of them are quite different... you can’t apply them to your own practice.” [referring to UpToDate]*

[14]—P7, Respiratory Staff Specialist.

Participants had varied opinions on how useable prescribing guidelines were. Some participants reported good usability with regard to access and navigation within the guidelines.

*“They’re all electronic now. So, I guess there’s pretty ready access as long as you’ve got a working computer.”*

—P5, AMS Pharmacist.

*“Yeah, I think the usability is fine. I think the sections are divided quite well. It’s easy to navigate, especially if you’ve been looking at it for quite some time.”*

—P3, AMS Pharmacist.

Other participants had issues with how guidelines fit into the patient care workflow due to electronic access issues and the large volume of information making it difficult to find answers quickly. Some participants also mentioned difficulty finding the correct information due to the need to use specific spelling and terminology.

*“I think technically, it is actually quite difficult, like it’s time consuming to access the Australian Therapeutic Guidelines or any other guidelines.”*

—P2, AMS Pharmacist.

*“Sometimes it can be a bit hard to find what you’re looking for. It can be a bit... and sometimes they’re slightly different advice in different guidelines.”*

—P11, Respiratory Staff Specialist.

There was disagreement about the comprehensiveness of the Therapeutic Guidelines, with some participants reporting they were too verbose to be used efficiently in practice, whilst others believed they were not comprehensive enough and did not adequately cover the treatment of more complex or unusual pathogens.

*“So not usable. They’re just put in so many words now and so many sections, you can’t find anything, so I think it’s really not user friendly anymore.”*

—P13, AMS Pharmacist.

*“You know, it’s not comprehensive, I guess in terms of the more unusual cases or atypical things.”*

—P17, Respiratory Staff Specialist.

### 2.3. Perceived Compliance with Antimicrobial Prescribing Guidelines

AMS pharmacists and physicians had different opinions on the level of compliance with prescribing guidelines. Prescribing guidelines here again refer to the Australian Therapeutic Guidelines unless otherwise specified [13]. AMS pharmacists perceived compliance to be suboptimal based on observations from practice and the results of prescribing audits.

*“I would say we definitely overtreat with the antibacterials. If I’m being generous, it’s probably about 50% compliant.”*

—P13, AMS Pharmacist.

Physicians generally believed their prescribing and that of their peers was appropriate.

*“I find it, especially at [my public hospital], the ED prescribing is very good. It’s very much in keeping with the guidelines. I haven’t seen anything unusual prescribed at all.”*

—P14, Respiratory Staff Specialist.

One physician highlighted the issue with lack of ownership over prescribing decisions and contributions to resistance.

*“I feel like I am guideline based for most of my prescribing… But if everyone takes that opinion, we aren’t ever going to change anything because everyone’s always going to say that ‘nope, my prescribing is great, it’s everybody else’s’, and that doesn’t get anyone anywhere.”*

—P11, Respiratory Staff Specialist.

There were some specific antimicrobials and conditions highlighted by AMS pharmacists where frequent inappropriate prescribing was observed. For example, using ceftriaxone and piperacillin–tazobactam where broad spectrum antimicrobials were not required, azithromycin for prolonged durations, and antibiotic use in viral infections.

*“Things like pneumonia… For some reason, I think Respiratory really just like to go straight to ceftriaxone regardless of the severity. And then as well, the macrolides, azithromycin sometimes gets prolonged more than 5 days.”*

—P1, AMS Pharmacist.

*“Our issues with respiratory [infections] would be in antibiotic use in infective exacerbation of COPD and COVID as well. A lot of them want to cover for a superimposed bacterial infection.”*

—P3, AMS Pharmacist.

There were specific situations where physicians mentioned intentionally deviating from guideline recommendations. Antimicrobials with simpler administration and less daily doses were preferred. Whilst ceftriaxone is only recommended for the treatment of severe CAP, some physicians reported using it in less severe cases due to its once-a-day administration via an intravenous push, rather than an intravenous infusion of benzylpenicillin four times a day. This was especially important in the private hospital setting or when patients were being treated by a community nursing service.

*“I will happily give them benzylpenicillin in the public hospital… Whereas it’s very variable with regard to nursing stuff in private hospitals… there is a bigger chance that the dose will be missed if the cannula doesn’t get put in. And so, because of that, in a private setting, I will always prefer ceftriaxone over benzylpenicillin.”*

—P14, Respiratory Staff Specialist.

This can also be the case for patients who require antimicrobials on discharge from hospital.

*“Medicines which are given twice a day versus things that are given three or four times a day starts to get confusing for people if they’re used to taking medicines only once a day versus other frequencies, that’s going to affect what I think is going to work for them. And so, I might deviate for that reason.”*

—P16, Infectious Diseases Staff Specialist.

Participants also reported deviating from the guidelines if their patient did not fit the guideline criteria. This was usually for patients with multiple simultaneous infections, severe infections, who were immunocompromised, had risk factors for resistance, or those in a critical condition where antimicrobials were used as salvage therapy.

*“It’s really just about kind of understanding whether the guidelines are applicable to the person in front of you. And knowing that the guidelines don’t cover all clinical scenarios or combinations of scenarios.”*

—P10, Respiratory Staff Specialist.

Physicians also highlighted that certain antimicrobials might be preferred for their non-infective properties.

*“I think the guidelines suggest doxycycline, but I tend to use azithromycin… that’s not just for its antimicrobial property, but I think that azithromycin has the best evidence for improving outcomes in community acquired pneumonia… because of its immunomodulatory effects.”*

—P10, Respiratory Staff Specialist.

### 2.4. Barriers to Optimal Antimicrobial Prescribing

**Figure 1:**
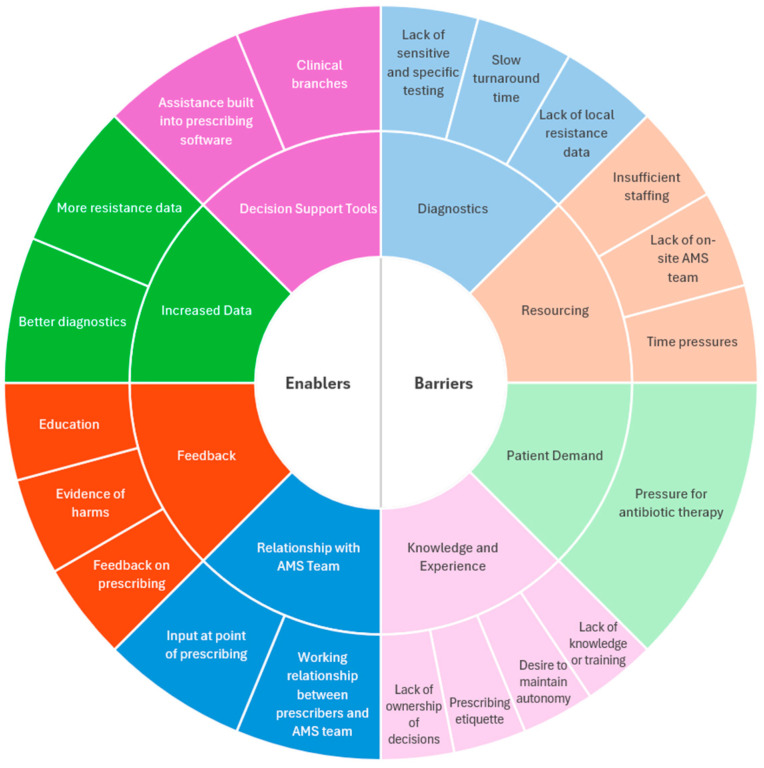
Barriers and enablers of optimal antibiotic prescribing in respiratory tract infections based on interviews with antimicrobial stewardship pharmacists and respiratory and infectious diseases physicians. A comparison of barriers and enablers between antimicrobial stewardship pharmacists, infectious diseases physicians, and respiratory physicians can be found in Supplement S4.

Participants discussed several barriers to optimal antimicrobial prescribing (Figure 1). Lack of sensitive and specific microbiological testing and slow turnaround time for results limited the use of cultures in acute infections. A lack of local resistance data to guide antimicrobial decisions was also reported.

*“And sputum cultures probably have little to no utility in managing acute pneumonia, both because of the way that those samples are handled by the micro lab and the time it takes for the results to come back.”*

—P16, Infectious Diseases Staff Specialist.

There were further diagnostic limitations discussed by participants from regional hospitals. Participants highlighted that the lack of on-site pathology, microbiology, and imaging prevented the use of directed therapy.

*“If a blood or any culture was done on Friday at 5pm, it doesn’t go to Sydney until Monday morning, and then it doesn’t get processed for however long that takes. So, it could be a full week before you have any results whatsoever… they’re flying blind most of the time just needing to use empirical therapy.”*

—P8, AMS Pharmacist.

Resourcing limitations after hours, in regional hospitals, private hospitals, and in community nursing teams resulted in broader-spectrum antimicrobials being prescribed due to lack of staffing for frequent administration or escalation in care. The relative lack of medical staff or locum staff in regional hospitals, private hospitals, and after hours in metropolitan hospitals resulted in broad spectrum antimicrobials to minimise risk of patient deterioration. In the private sector, physicians may not review their patients in person every day, and antimicrobials would generally be continued between reviews regardless of the number of days between ward rounds.

*“Private hospitals are not good places for very sick people, because there’s less doctors on the wards… so if something does go wrong, it is often picked up later in a private hospital. And so, you tend to play it safer in a private hospital and you’d use more intravenous antibiotics for longer.”*

—P14, Respiratory Staff Specialist.

Participants from regional hospitals and private hospitals stated they often did not have an on-site AMS team, there were fewer antimicrobial specialists available for advice, and there was less oversight on antimicrobial prescribing patterns within the hospital.

*“Because we don’t have an ID physician on site who can give them a tap on the shoulder and say, “Hey, the guidelines say this but you are doing this, why is that?” kind of thing. We don’t have that manpower to kind of enforce that.”*

—P4, AMS Pharmacist.

A few participants highlighted that patient demand for antimicrobial therapy could promote suboptimal prescribing. It was reported to stem from overprescribing in the community, private patients believing they have more of a say in their care, and patient perception that if they were sick enough to be in hospital they were sick enough for intravenous antimicrobials.

*“One barrier is patient expectations... They want a script for something, and, you know, they expect that from their GP, they expect that from you. And it can be challenging to combat that in many ways. And that’s something that’s been happening for a long time. People can equate cough and sputum with infection.”*

—P17, Respiratory Staff Specialist.

There was a perception amongst some AMS pharmacists and infectious diseases staff specialists that physicians may not have sufficient knowledge of antimicrobials.

*“It depends on the prescriber and their awareness of the issue of antimicrobial resistance and the issue of spectrum. And some doctors may just prescribe broader just because they think that that’s likely to quickly get on top of the problem.”*

—P12, Infectious Diseases Staff Specialist.

Furthermore, physicians who were trained overseas may not have adequate AMS training or knowledge of local prescribing recommendations.

*“A lot of overseas doctors, whose knowledge may not be as good... and that makes it more difficult as well.”*

—P15, Respiratory Staff Specialist.

Participants discussed a lack of ownership of antimicrobial decisions and unwillingness to change antimicrobial therapy commenced by another physician.

*“I think within the hospital itself, we often stumble across people who are on what we think is too broad, and we’re trying to narrow them down, but sometimes there’s a reticence if they’re on a pathway, and they probably could have gotten away with a narrow spectrum, but they’re improving, and maybe you don’t then narrow them back down.”*

—P17, Respiratory Staff Specialist.

There was also a resistance to change, and a preference to maintain autonomy and prescribing habits rather than following guidelines, discussed by some of the AMS pharmacists.

*“But where there’s resistance to change, this tends to be what is the reasoning behind the resistance to following guidelines.”*

—P9, AMS Pharmacist.

Finally, AMS pharmacists stated that intense workloads minimised time for physicians to access antimicrobial guidelines at the point of prescribing to assist in decision-making.

*“Freestyle prescribing is a lot of the problem too, because it relies on the clinicians to either have the guidelines in front of them on a different page or use their memory. Most of the time, because they’re so busy, they’re using their memory and their memory is not great for every single respiratory tract infection.”*

—P8, AMS Pharmacist.

### 2.5. Enablers for Optimal Antimicrobial Prescribing

Participants discussed several enablers for optimal prescribing (Figure 1). Firstly, most AMS pharmacists and some physicians mentioned that a working relationship between physicians and the AMS team can have a positive influence on prescribing through greater acceptance of AMS interventions and involvement in projects aimed at optimising prescribing.

AMS pharmacists also reported having input at the point of prescribing to be incredibly beneficial, as there was a low likelihood of changing antimicrobial therapy after the course had started.

*“In our ED AMS rounds we try to focus and see those CAP [community-acquired pneumonia] patients that come in just to intervene at the point of diagnosis as well, so getting the antibiotic right from the beginning. There’s been studies shown as well that demonstrates when antibiotics are started in ED, that they’re unlikely to change on the ward, they just get continued through.”*

—P3, AMS Pharmacist.

Some participants reported providing feedback on prescribing habits, and comparing these to peers, would be useful in causing a reflection on practices.

*“The other thing that sometimes works is to have anonymised feedback, so that I get my feedback about what I prescribed… and that is compared to all of my peer colleagues at my hospital… That would be a way of providing feedback to people that’s individualised and anonymised about where their practice sits with reference to everyone else in their peer hospital.”*

—P11, Respiratory Staff Specialist.

Providing feedback and evidence on the harms of inappropriate prescribing was also reported as a strategy to improve antimicrobial prescribing by some AMS pharmacists.

*“And so now I’m trying to like give evidence. You know, this practice actually has caused C diff [Clostridioides difficile] in this case and giving 72 h of cefazolin on all knee replacement is actually increasing your length of stay. So, I’m trying to use other angles to try and say “Well, like, you’re actually harming people in other ways”.”*

—P2, AMS Pharmacist.

Increased access to diagnostics, particularly in regional settings, and access to local resistance data was reported to enable informed decision-making around antimicrobials.

*“If the lab… published on a regular basis… the amount of antimicrobial resistance noted in pneumococcus, Haemophilus… then people could start to make decisions based on evidence, on science, on what’s in the local community.”*

—P11, Respiratory Staff Specialist.

Education to physicians and to empower other pharmacists was an enabler mentioned by AMS pharmacists.

*“A lot of education and re-education. I would say, there’s a lot of doctors obviously rotating through and whatnot, and sometimes the Advanced Trainees or the Basic Physician Trainees might not review the antibiotics, or just continue as per consultant. But if we continue to re-educate, maybe they can also flag it. Having a top-down approach as well.”*

—P3, AMS Pharmacist.

Several decision support tools were described by participants to guide optimal prescribing. These included having antimicrobial guidelines, prescribing prompts, and infection risk assessments built into prescribing software.

Clinical branches were also a method suggested that could direct decisions in a more efficient way than reading text-based guidelines.

*“You say “I’m treating CAP”, and then it would tell you, “These are your options for CAP”, and you would select. Does your patient have bilateral chest x-ray changes? Does your patient have a penicillin allergy? Does your patient have oxygen saturations less than 90%? And blood pressure? And then it kind of goes down the algorithm of your patient likely has severe pneumonia. You should chart this and this and do these tests.”*

—P6, AMS Pharmacist.

## 3. Discussion

This study demonstrates that AMS pharmacists and physicians primarily use national prescribing guidelines to inform their antimicrobial choices. There is variability in compliance with these guidelines due to the difficulty implementing them within the busy workflow of patient care, their lack of information in complex infections, and the inability to account for the complexities and pragmatics of treating real patients. Whilst some deviation from guidelines is expected as a result of these, compliance with prescribing guidelines in most cases is vital to minimise contributions to resistance. Understanding the resources used to aid decision-making and barriers and enablers to guideline adherence and optimal antibiotic use is required for the successful design, uptake, and implementation of quality improvement interventions. This study provides this insight and also provides a valuable discussion on issues specific to under-represented private and regional hospitals.

Barriers to optimal prescribing include diagnostic limitations, staff and resourcing limitations, patient demand, lack of knowledge and willingness to change, and time pressures. Strategies to enable optimal prescribing included a positive working relationship between physicians and AMS teams, providing feedback on prescribing habits and patient outcomes, equity of access to diagnostics, education, and decision support tools incorporated within prescribing software. Novel barriers to guideline-based prescribing identified in this study include a lack of sensitive, specific, and rapid diagnostics for RTIs, hospital staffing and resourcing limitations, time pressures, and individual acknowledgement and ownership of contribution to AMR. Novel enablers include physicians’ relationship with the AMS team, providing evidence of the harms of suboptimal prescribing, and decision support tools.

Most participants used national evidence-based guidelines to support their prescribing. Whilst participants were generally in agreement with the recommendations in these guidelines, there were dissonant views on how useful the guidelines are in practice. Some participants found them helpful and easy to access, whilst others found them impractical given their length and challenging navigation. Most physicians self-reported their prescribing to be mostly appropriate and guideline-based, in contrast to reported AMS pharmacist observations and national audit results [1] showing high rates of non-compliance consistent with previous studies [15]. Opposing views on compliance between physicians and AMS pharmacists can be partly explained by the value physicians place on clinical and personal experience and the acceptance of guideline non-compliance if recommendations do not align with their personal views [16]. Additionally, it may also be due to social desirability bias when participating in a research study. Intentions to follow guidelines do not always correlate with guideline adherence [17]. This is likely due to the numerous factors clinicians must balance when making decisions that guidelines do not necessarily account for [18,19]. There are competing approaches for evidence-based versus bedside medicine [20], and deviations from guidelines may be justified in complex cases to simplify administration or to utilise the immunomodulatory properties of certain antibiotics. The most notable discrepancies reported were in the use of broader-spectrum antibiotics for longer durations than were recommended in guidelines. This was consistent with the general approach to ‘err on the side of caution’ as previously documented [20,21]. As identified in this study, acknowledgement and ownership of antibiotic decisions and contributions to resistance will be important in improving prescribing and addressing resistance.

Understanding the barriers and enablers to optimal antibiotic prescribing has implications for the development of future interventions. The World Health Organization highlights the importance of diagnostic stewardship for robust microbiological diagnosis and directed antibiotic therapy [22]. However, diagnostics for RTIs are especially poor, with previous work echoing results from participants that they lack the sensitivity, specificity, and speed needed to be useful in acute infections [23]. These limitations are more pronounced in regional hospitals where imaging facilities are not always available and the turnaround time for microbiology and pathology can be incredibly delayed. Funding point-of-care diagnostics that can identify pathogens or promptly report procalcitonin levels may be useful [24,25,26]. The COVID-19 pandemic saw a rapid escalation in point-of-care diagnostics for identifying viral pathogens; however, those available to diagnose bacterial infections are still not widely used due to time pressures incorporating these into the usual workflow, reliability, and high cost [24,27,28]. Furthermore, investing resources into developing updated local antibiograms can make prescribing more evidence-based where clinicians do not have to overprescribe due to concerns about resistance rates. Insufficient time and staffing impede prudent antibiotic prescribing [29]. Unfortunately, these issues are unlikely to resolve within the current health system, and clinicians will continue to be required to balance resources dedicated to AMS with all their other clinical and non-clinical priorities. Decision support tools at the point of prescribing may be one way to address this by streamlining and simplifying the decision-making process [30,31]. Finally, poor knowledge on antimicrobials and recommendations in guidelines may have negative consequences on antibiotic appropriateness. Physicians generally self-report their antibiotic use to be optimal; however, perceptions from the study’s AMS pharmacists and from knowledge assessments in the literature are not in agreement [32]. Physicians also state they rarely review guidelines and instead rely on their knowledge of those guidelines and clinical experience. Familiarity and complacency in accessing guidelines may result in recall bias and out-of-date information [11]. Ways of improving appropriate prescribing may include a regular interactive multi-faceted educational approach to physicians [33] and providing feedback on prescribing habits [5,34]. Patient education to increase knowledge on the utility of antibiotics in viral infections can reduce demand and pressure [35]. Therefore, addressing these barriers and considering these enablers when designing future AMS interventions is vital.

This study has some limitations. Firstly, only senior physicians were interviewed, and these views may not represent those of their junior colleagues. However, the medical hierarchy and prescribing etiquette often means that the prescribing of junior physicians is influenced by their seniors. Secondly, self-reporting and social desirability bias may have influenced the results, particularly around guideline compliance; however, the purpose of this study was to gain insights into perceptions of compliance—not to quantitatively measure rates of compliance. Guideline compliance of antimicrobials prescribed in Australian hospitals has already been well-reported in national audits [1]. There was a relatively small number of interview participants in certain subgroups such as regional hospitals, private hospitals, and infectious diseases clinicians, and their views may not be generalisable. Finally, interview participants were limited to one state in Australia, and barriers and enablers may not necessarily be extrapolated to other settings.

## 4. Materials and Methods

The methods for this study followed the consolidated criteria for reporting qualitative research (COREQ) guidelines (Supplement S2) [36]. This paper is a component of a larger study and part of the data has already been reported [under review] [19]. The results discussed in this paper are distinct.

### 4.1. Ethical Considerations

Ethical approval was granted by the Western Sydney Local Health District Human Research Ethics Committee (2023/ETH01053). Written consent from all participants was obtained, recorded, and stored in the REDCap data collection tool (version 13.10.4) prior to interviews commencing [37,38].

### 4.2. Setting

This study was conducted in New South Wales, Australia. Participants included AMS pharmacists and respiratory and infectious diseases staff specialists from public and private, metropolitan, and regional hospitals across the state. All hospitals used electronic prescribing systems.

### 4.3. Sampling and Recruitment

Participants were recruited through their professional organisations, the researchers’ networks, and via passive snowballing. A purposive sampling strategy was employed to ensure diversity demographic characteristics and clinical backgrounds. Recruitment continued until thematic saturation was achieved.

### 4.4. Data Collection

Qualitative data was collected through semi-structured interviews with AMS pharmacist and physicians. Interviews explored opinions on antimicrobial recommendations in evidence-based guidelines, perceived level of adherence to prescribing guidelines, and barriers and enablers to optimal antimicrobial use in RTIs.

Two separate semi-structured interview guides, one for pharmacists and one for physicians, were developed (Supplement S3). Interview guides were piloted with three pharmacists and one respiratory physician prior to the interviews commencing. Feedback was obtained on the length of the interview, flow and wording of the questions, as well as whether the pilot participants could understand the meaning of the questions and whether the questions adequately addressed the study aims. Minimal adjustments were made after piloting. Interviews were conducted virtually and recorded via Zoom or Microsoft Teams and transcribed verbatim using Otter.ai transcription software (2024) [39].

Demographic data, including the participant’s current role, hospital characteristics, and years of experience, were collected during the interviews.

### 4.5. Data Analysis

The interview transcripts were examined inductively following Braun and Clarke’s thematic analysis approach [40]. A coding framework was developed, and a second independent coder assessed 20% of the transcripts to ensure reliability and accuracy. Interviews were coded using NVIVO 13 qualitative analysis software [41].

## 5. Conclusions

This study broadens the scope of research on the use of guidelines and barriers and enablers of optimal antibiotic prescribing in hospitalised patients with RTIs. Clinicians were aware of relevant guidelines but did not always follow their recommendations due to difficulty incorporating them at the point of prescribing, lack of data on rare pathogens or conditions, and a desire to rely more on clinical experience. Suboptimal antibiotic prescribing is a result of inadequate diagnostics and staffing, patient treatment pressure, and lack of knowledge and ownership of the impact of prescribing decisions. Creating a positive relationship between the AMS team and physicians, prescribing feedback, and increasing the availability of data and decision support tools to guide prescribing can assist in overcoming some of these barriers. The impact of these barriers on prescribing behaviour must be addressed in AMS programs. Focusing on the enablers and incorporating these into future prescribing interventions can optimise prescribing and reduce the harms of inappropriate antibiotic use.

## Figures and Tables

**Table 1:** Coding framework with themes and subthemes.

Theme	Subtheme	Description of Subtheme
Resources used to guide antimicrobial decisions	Nationally endorsed guidelines (TGs)	Participants’ discussion of using the Therapeutic Guidelines (Australian-based therapeutic consensus guidelines) to guide antimicrobial decisions in respiratory tract infections.
	Local facility policies and procedures	Hospital or health-district specific guidelines that either replaced national guidelines or were to be used alongside national guidelines for specific conditions.
	Other guidelines used when national guidelines insufficient	Guidelines used by participants in specific situations when the Therapeutic Guidelines were insufficient.
	Specialist AMS, ID or microbiology clinicians	Consulting with antimicrobial specialists for expert opinions.
Healthcare professionals’ opinions on antimicrobial guidelines	Applicability of recommendations	Participants’ discussion of how applicable the recommendations in the Therapeutic Guidelines were to their patients.
	Usability of guidelines in practice	Participants’ discussion of how usable the Therapeutic Guidelines were in practice.
Perceived compliance with antimicrobial prescribing guidelines	Variation in perceived compliance between AMS pharmacists and physicians	Discussion of the perceived level of compliance with antimicrobial prescribing guidelines.
	Specific antimicrobials/infections	Specific antimicrobials or infections discussed by AMS pharmacists that had high rates of inappropriate prescribing.
	Specific situations requiring deviation from guidelines	Specific situations where physicians mentioned intentionally deviating from guideline recommendations.
	Variation in perceived compliance between AMS pharmacists and physicians	Discussion of the perceived level of compliance with antimicrobial prescribing guidelines.
Barriers to optimal prescribing	Diagnostic limitations in RTIs	Participants’ discussion of limitations with the state of current diagnostics available to guide antimicrobial choices.
	Resource and staffing limitations	Participants’ discussions of how resourcing within medical and nursing teams impacted antimicrobial decisions.
	Healthcare setting	Participants from private and regional hospitals described how their setting impacted antimicrobial prescribing.
	Patient demand	Patient demand for antimicrobial therapy could impact antimicrobial decisions.
	Clinical experience and knowledge	Physicians’ clinical experience, knowledge, and autonomy influenced their decisions.
	Time pressures	Time constraints impacted the ability to perform thorough patient and guideline assessment.
Enablers of optimal prescribing	Relationship between physician and AMS team	How the working relationship between physicians and the AMS team impacted antimicrobial choices.
	Provide feedback on prescribing habits	Provide feedback to physicians on their prescribing habits and compare these to others in their team or hospital.
	Providing evidence of harms of suboptimal prescribing	Provide evidence of harms of inappropriate prescribing on the patient and health system.
	Increased access to data and diagnostics	How access to antimicrobial data and diagnostics informed antimicrobial decisions.
	Education	Education of physicians and other healthcare professionals on AMS.
	Decision support tools	Antimicrobial decision support tools built-in to prescribing software to guide optimal decisions.

## Data Availability

Additional data can be found in the Supplementary Materials.

## Supplementary Materials

The following supporting information can be downloaded at https://www.mdpi.com/article/10.3390/antibiotics14101039/s1, Supplement S1: Coding framework with quotes; Supplement S2: COREQ criteria; Supplement S3: Interview guides; Supplement S4: Barrier and enabler comparison.

## References

[1] Royal Melbourne Hospital and the National Centre for Antimicrobial Stewardship (2024). Antimicrobial Prescribing Practice in Australian Hospitals. Results of the 2022 Hospital National Antimicrobial Prescribing Survey.

[2] Australian Commission on Safety and Quality in Health Care (2023). AURA 2023: Fifth Australian Report on Antimicrobial Use and Resistance in Human Health.

[3] Stojanovic Z., Gonçalves-Carvalho F., Marín A., Abad Capa J., Domínguez J., Latorre I., Lacoma A., Prat-Aymerich C. (2022). Advances in diagnostic tools for respiratory tract infections: From tuberculosis to COVID-19–changing paradigms?. ERJ Open Res..

[4] World Health Organization (2015). Global Action Plan on Antimicrobial Resistance.

[5] Davey P., Marwick C.A., Scott C.L., Charani E., McNeil K., Brown E., Gould I.M., Ramsay C.R., Michie S. (2017). Interventions to improve antibiotic prescribing practices for hospital inpatients. Cochrane Database Syst. Rev..

[6] McDonagh M.S., Peterson K., Winthrop K., Cantor A., Lazur B.H., Buckley D.I. (2018). Interventions to reduce inappropriate prescribing of antibiotics for acute respiratory tract infections: Summary and update of a systematic review. J. Int. Med. Res..

[7] Chuki P., Dorji T., James R., Wangchuk K., Yangzom S., Dema Y., Wangchuk S., Wangdi D., Deki T., Limbu C. (2023). Antibiotic use and quality indicators of antibiotic prescription in Bhutan: A point prevalence survey using the Australian National Antimicrobial Prescribing Survey tool. JAC-Antimicrob. Resist..

[8] Canadian Antimicrobial Resistance Surveillance System (CARSS) (2024). National Antimicrobial Prescribing Survey. https://health-infobase.canada.ca/carss/amu/results.html?ind=02.

[9] Saliba-Gustafsson E.A., Nyberg A., Borg M.A., Rosales-Klintz S., Stålsby Lundborg C. (2021). Barriers and facilitators to prudent antibiotic prescribing for acute respiratory tract infections: A qualitative study with general practitioners in Malta. PLoS ONE.

[10] O’Doherty J., Leader L.F.W., O’Regan A., Dunne C., Puthoopparambil S.J., O’Connor R. (2019). Over prescribing of antibiotics for acute respiratory tract infections; a qualitative study to explore Irish general practitioners’ perspectives. BMC Fam. Prac..

[11] Sedrak A., Anpalahan M., Luetsch K. (2017). Enablers and barriers to the use of antibiotic guidelines in the assessment and treatment of community-acquired pneumonia—A qualitative study of clinicians’ perspectives. Int. J. Clin. Pract..

[12] Broom J.K., Broom A.F., Kirby E.R., Gibson A.F., Post J.J. (2017). Clinical and social barriers to antimicrobial stewardship in pulmonary medicine: A qualitative study. Am. J. Infect. Control.

[13] Therapeutic Guidelines Limited (2025). Therapeutic Guidelines. Melbourne. https://www.tg.org.au.

[14] Connors R.F. UpToDate. Wolters Kluwer. https://www.wolterskluwer.com/en-au/solutions/uptodate.

[15] Almatar M.A., Peterson G.M., Thompson A., McKenzie D.S., Anderson T.L. (2015). Community-acquired pneumonia: Why aren’t national antibiotic guidelines followed?. Int. J. Clin. Pract..

[16] Charani E., Castro-Sanchez E., Sevdalis N., Kyratsis Y., Drumright L., Shah N., Holmes A. (2013). Understanding the determinants of antimicrobial prescribing within hospitals: The role of “prescribing etiquette”. Clin. Infect. Dis..

[17] Cortoos P.-J., Schreurs B.H.J., Peetermans W.E., De Witte K., Laekeman G. (2012). Divergent Intentions to Use Antibiotic Guidelines: A Theory of Planned Behavior Survey. MDM.

[18] McKay R., Mah A., Law M.R., McGrail K., Patrick D.M. (2016). Systematic Review of Factors Associated with Antibiotic Prescribing for Respiratory Tract Infections. Antimicrob. Agents Chemother..

[19] Reali S., Cho J.-G., Alffenaar J.-W., Aslani P. (2025). Factors Influencing Antimicrobial Decision-Making in Respiratory Tract Infections. Resp. Med..

[20] Wojcik G., Ring N., McCulloch C., Willis D.S., Williams B., Kydonaki K. (2021). Understanding the complexities of antibiotic prescribing behaviour in acute hospitals: A systematic review and meta-ethnography. Arch. Public Health.

[21] Reali S., Kwang Y.C., Cho J.-G., Alffenaar J.-W., Aslani P. (2025). Factors influencing physicians’ antimicrobial prescribing decisions: A systematic review of qualitative studies. BJCP.

[22] World Health Organization (2016). Diagnostic Stewardship: A Guide to Implementation in Antimicrobial Resistance Surveillance Sites.

[23] Caliendo A.M., Gilbert D.N., Ginocchio C.C., Hanson K.E., May L., Quinn T.C., Tenover F.C., Alland D., Blaschke A.J., Bonomo R.A. (2013). Better Tests, Better Care: Improved Diagnostics for Infectious Diseases. Clin. Infect. Dis..

[24] Basharat S., Horton J., Emerging Point-of-Care Tests for Differentiating Bacterial and Viral Infections (2021). CADTH Horizon Scan, Canadian Agency for Drugs and Technologies in Health. https://www.ncbi.nlm.nih.gov/books/NBK594330.

[25] Australian Commission on Safety and Quality in Health Care (2023). Antimicrobial Stewardship in Australian Health Care.

[26] Lindström J., Nordeman L., Hagström B. (2015). What a difference a CRP makes. A prospective observational study on how point-of-care C-reactive protein testing influences antibiotic prescription for respiratory tract infections in Swedish primary health care. Scand. J. Prim. Health Care.

[27] Gentilotti E., De Nardo P., Cremonini E., Górska A., Mazzaferri F., Canziani L.M., Hellou M.M., Olchowski Y., Poran I., Leeflang M. (2022). Diagnostic accuracy of point-of-care tests in acute community-acquired lower respiratory tract infections. A systematic review and meta-analysis. Clin. Microbiol. Infect..

[28] Jamshidi N., Waine M., Binet M., Mohan V., Carter D.J., Morgan B. (2024). The adoption of point of care testing technologies for respiratory tract infections in primary care in Australia: Challenges and facilitators. Diagn. Microbiol. Infect. Dis..

[29] Borek A.J., Anthierens S., Allison R., McNulty C.A.M., Anyanwu P.E., Costelloe C., Walker A.S., Butler C.C., Tonkin-Crine S., on behalf of the STEP-UP Study Team (2020). Social and Contextual Influences on Antibiotic Prescribing and Antimicrobial Stewardship: A Qualitative Study with Clinical Commissioning Group and General Practice Professionals. Antibiotics.

[30] MacFadden D.R., Daneman N. (2023). Can Decision Support Tools Improve Empiric Antibiotic Prescribing?. NEJM Evid..

[31] Nabovati E., Jeddi F.R., Farrahi R., Anvari S. (2021). Information technology interventions to improve antibiotic prescribing for patients with acute respiratory infection: A systematic review. Clin. Microbiol. Infect..

[32] Md Rezal R.S., Azmi H.M., Alrasheedy A.A., Fahad S., Aryani M.Y.F., Godman B. (2015). Physicians’ knowledge, perceptions and behaviour towards antibiotic prescribing: A systematic review of the literature. Expert Rev. Anti-Infect. Ther..

[33] Arnold S.R., Straus S.E. (2005). Interventions to improve antibiotic prescribing practices in ambulatory care. Cochrane Database Syst. Rev..

[34] Langdridge D., Virhia J., McMullan R., Banks D., Biard O., Charitonos K., Alunyo J.P., Kagoya E.K., Olupot-Olupot P. (2024). Effectiveness of work-based educational interventions for antimicrobial stewardship: A systematic review. JAC-Antimicrob. Resist..

[35] Hunter C.R., Owen K. (2024). Can patient education initiatives in primary care increase patient knowledge of appropriate antibiotic use and decrease expectations for unnecessary antibiotic prescriptions?. Fam. Pract..

[36] Tong A., Sainsbury P., Craig J. (2007). Consolidated criteria for reporting qualitative research (COREQ): A 32-item checklist for interviews and focus groups. Int. J. Qual. Health Care.

[37] Harris P.A., Taylor R., Minor B.L., Elliott V., Fernandez M., O’Neal L., McLeod L., Delacqua G., Delacqua F., Kirby J. (2019). The REDCap consortium: Building an international community of software platform partners. J. Biomed. Inform..

[38] Harris P.A., Taylor R., Thielke R., Payne J., Gonzalez N., Conde J.G. (2009). Research electronic data capture (REDCap)—A metadata-driven methodology and workflow process for providing translational research informatics support. J. Biomed. Inform..

[39] AISense Inc (2025). Otter.ai. https://otter.ai.

[40] Braun V., Clarke V. (2006). Using thematic analysis in psychology. Qual. Res. Psychol..

[41] Lumivero (2020). NVivo 13 [Computer Software]. https://lumivero.com/products/nvivo/.

