# Supplementary material for: Barriers and Enablers to Optimal Antimicrobial Use in Respiratory Tract Infections
Source: Antibiotics (Basel). 2025 Oct 16;14(10):1039. doi: 10.3390/antibiotics14101039 (PMC12562221; doi:10.3390/antibiotics14101039)
Supplement: Supplementary file 1 [file antibiotics-14-01039-s001.zip › Supplement 1 - Coding Framework with Quotes.pdf]

Theme	Subtheme	Description of Subtheme	Quotes
Resources used to guide antimicrobial decisions	Nationally endorsed guidelines (eTGs)	Participants discussion of using the Therapeutic Guidelines (Australian-based therapeutic consensus guidelines) to guide antimicrobial decisions in respiratory tract infections.	"Mainly the eTG guideline. Someone smarter than me has sat on the eTG guidelines and argued the toss about what antibiotics to use. And they've presumably looked at the Australian data and they're smarter than me, they know more than me, so I may as well just follow what they say." P11<br><br>"I use eTG. That's sort of my preferred guideline." P17<br><br>"Most of the time Therapeutic Guidelines. That's our overarching guideline." P8
	Local facility policies and procedures	Hospital or health-district specific guidelines that either replaced national guidelines or were to be used alongside national guidelines for specific conditions.	"We are developing hospital guidelines for IV to oral switch or IV to oral step down. So that's applicable to respiratory tract infections." P1<br><br>"The community acquired pneumonia one... I think it just has an extra section for if patients had been on antibiotics in the community before coming into hospital." P5<br><br>"So, generally the population in Western Sydney local health district are deemed to perhaps come from a more migrant background. So, a lot of these patients may have lived overseas and have migrated recently. So, they're considered to be at high risk of multi-drug-resistant organisms. And we've got a large group of CF patients as well. So, I guess the acuity of the district sort of drove the need to have a local guideline which might recommend more broader antibiotics." P3<br><br>"In an effort to try and curb ceftriaxone use throughout our district, we do have a hospital health pathway for community acquired pneumonia, hospital acquired pneumonia, sepsis, and infective exacerbations of. So, they steer clear based on our local antibiograms, they steer clear of jumping to ceftriaxone for more severe infections and using high dose benzylpenicillin and gentamicin." P8<br><br>"A lot of the COVID management is probably influenced about what we do in this institution, which differs from other institutions." P16

			“And then then we have our CF Manual Handbook.” P7
	Other guidelines used when national guidelines insufficient	Guidelines used by participants in specific situations when the Therapeutic Guidelines was insufficient.	“And it would be a rare occasion that I have to like consult, you know, the literature or something. Maybe it's like I found a weird organism or something like that.” P10<br><br>“So, you can look up the Australian Medicines Handbook, for instance, and use their tools for drug-drug interactions, particularly with antifungal agents, the CYP3A4 inhibitors. And then the TB drugs rifampicin, which we also use for MRSA. Rifampicin is a very strong inducer.” P12<br><br>“For TB, it's largely going to be WHO endorsed therapy, which, again, we sometimes have to customise based on adverse events or more resistance profiles. For NTM bronchiectasis, again, I'd follow the International Society endorsed guidelines that come out of Europe and the US or British guidelines. So hence, the individual disease entity.” P16<br><br>“I find Therapeutic Guidelines, is really... it's got a good kind of like summary of how we should be treating them. But I find that it doesn't include all indications. So that's why I'm using UpToDate. For the more, I guess, complex patient or where the information is lacking in the Therapeutic Guidelines.” P4
	Specialist AMS, ID or microbiology clinicians	Consulting with antimicrobial specialists for expert opinions.	“I'll, you know, often share that decision making with some of my Infectious Diseases colleagues when it's tricky.” P11<br><br>“Yes, if patients definitely got complicated infection, then we always have an Infectious Disease consult or speak to AMS for approval of antibiotics.” P7<br><br>“Often with really sick ICU patients, we will get [AMS team] involved at the outset. Because as you know, those ones really can be quite difficult. It can be important in a very time sensitive way to get it right early.” P17
Healthcare professionals’ opinions on	Applicability of recommendations	Participants discussion of how applicable the recommendations in the	“Applicability in like most cases, it's a pretty good match.” P8<br>“I'm not the biggest fan of their suggestion of IV azithromycin in severe pneumonia, because I don't think anyone ever needs IV azithromycin unless

antimicrobial guidelines		Therapeutic Guidelines were to their patients.	they're completely nil by mouth. So that's just probably a minor little irk that I have." P13<br><br>"I've had a few patients come through with mycoplasma these days, which seems to be not always responsive to the azithromycin." P14<br><br>"I do look it Up to Date. Sometimes just to see people's approach to things, but I must say, it's not always referable to our setting." P17<br><br>"Because you do notice some differences in the availability of the drugs or the drug to go to. So, you try to use the same class of drug where there's aminoglycosides or other things. Some of them are applicable but some of them are quite different, like in the US and things like that. So, you would kind of not apply them, I guess you can't apply them to your own practice." P7
	Usability of guidelines in practice	Participants discussion of how usable the Therapeutic Guidelines were in practice.	"I think the thing that I like the most about them is that they actually give some rationale." P10<br><br>"But I think they're reasonably functional and usable. They cover most topics fairly well." P15<br><br>"Yeah, I the usability is fine. I think the sections are divided quite well. It's easy to navigate, especially if you've been looking at it for quite some time." P3<br><br>"They're all electronic now. So, I guess there's pretty ready access as long as you've got a working computer." P5<br><br>"Sometimes it can be a bit hard to find what you're looking for. It can be a bit… and sometimes they're slightly different advice in different guidelines." P11<br><br>"So not usable. They're just put in so many words now and so many sections, you can't find anything, so I think it's really not user friendly anymore." P13<br><br>"You know, it's not comprehensive, I guess in terms of the more unusual cases or atypical things." P17

			“I think technically, it is actually quite difficult, like it's time consuming to access the Australian Therapeutic Guidelines or any other guidelines.” P2<br><br>“Technically, it's wrong. They've got a forced reflow on their page at the moment… like when you click on something, it just spins forever. Sometimes when you search, it takes you back to the homepage for no reason.” P2<br><br>“You have to know the terminology. And sometimes the names of infections can be slightly different. So, you have to know what to look up, I guess, and know your way around the guidelines. The Therapeutic Guidelines is great, but there are a lot of chapters, and then a lot of things within that, and if you do a search at the top box… it comes up with lots of different things, so it can take a bit of time to find the right spot in the guideline.” P5<br><br>“Sometimes some comments are quite vague, which then makes it quite tricky for the clinical pharmacists to then make judgment on or even to guide to make any recommendations in those greyer areas.” P9
Perceived compliance with antimicrobial prescribing guidelines	Variation in perceived compliance between AMS pharmacists and physicians	Discussion of the perceived level of compliance with antimicrobial prescribing guidelines.	“I feel like my prescribing is pretty good. And I feel like I am guideline based for most of my prescribing… But if everyone takes that opinion, we aren’t ever going to change anything because everyone's always going to say that 'nope, my prescribing is great, it's everybody else's', and that doesn't get anyone anywhere.” P11<br><br>“I find it, especially at [my public hospital], the ED prescribing is very good. It's very much in keeping with the guidelines. I haven't seen anything unusual prescribed at all.” P14<br><br>“I would say we definitely overtreat with the antibacterials. If I'm being generous, it's probably about 50% compliant.” P13<br><br>“Generally, you know, based on our AMS round they are generally not that good. So, you know, when I looked at the data for the AMS round, the top

			inappropriate use of antibiotic, in terms of specialty, respiratory is always one of the top 3." P1<br><br>"I would say, going off the data that we have off NAPS, it's done poorly... There's over-broadening of spectrum, durations longer than required, and sometimes the dosing... But, yeah, look, I can't say that most prescriptions meet Therapeutic Guidelines." P9
	Specific antimicrobials/infections	Specific antimicrobials or infections discussed by AMS pharmacists that had high rates of inappropriate prescribing.	"So, things like pneumonia... For some reason, they, I think respiratory really just like to go straight to ceftriaxone regardless of the severity. And then as well, the macrolides as well, azithromycin sometimes gets prolonged more than 5 days." P1<br><br>"But most of the inappropriate use of antimicrobials is mainly in those pneumonia, bronchiectasis, infective exacerbation of COPD." P1<br><br>"There's over-broadening of spectrum, durations longer than required, and sometimes the dosing." P9<br><br>"It's more so, our issues with respiratory would, I would say, be in antibiotic use in infective exacerbation of COPD, COVID as well, a lot of them want to cover for a superimposed bacterial infection, and in CAP, we're working on trying to improve prescribing there." P3<br><br>"I would say I intervene a lot more on say Tazocin [piperacillin-tazobactam] for respiratory tract infections. Most of my interventions are on azithromycin for respiratory tract infections. A lot of my interventions are also on 3rd generation cephalosporins, ceftriaxone and cefotaxime. Those are the main ones I see for respiratory tract infections. I do have to say I intervene a bit less on Augmentin only because by the time I see them, it's not a restricted agent in our hospital, but I do think it's overprescribed a lot of the time. And that would be for both IV and oral actually, yeah." P6
	Specific situations requiring deviation from guidelines	Specific situations where physicians mentioned intentionally deviating from	"You know, medicines which are given twice a day versus things that are given three or four times a day starts to get confusing for people if they're used to taking medicines only once a day versus other frequencies, that's going to

		guideline recommendations.	affect what I think is going to work for them. And so, I might deviate for that reason. I mean, I just think that that's something that the guidelines often just sort of smooth over without really considering, and the reality is that you need to just be mindful that that's a real-life factor. The pragmatics of it." P16<br><br>"I will happily give them benzylpenicillin in the public hospital... the nurses will know how important the antibiotics are, and we'll get that cannula in quickly. Whereas it's very variable with regard to nursing stuff in private hospitals... there is a bigger chance that the dose will be missed if the cannula doesn't get put in. And so, because of that, in a private setting, I will always prefer ceftriaxone over benzylpenicillin. Whereas in a public setting, I'm much more happy to use benzylpenicillin." P14<br><br>"I mean, I guess it's really just about kind of understanding whether the guidelines are applicable to the person in front of you. And knowing that the guidelines don't cover all clinical scenarios or combinations of scenarios that you… like, I just gave the example of the COPD exacerbation. There's nothing in the guidelines that says what to do when you have a COPD patient with an exacerbation and suspected community acquired pneumonia +/- bacteraemia." P10<br><br>"But one has to also remember that they are just guidelines, and sometimes the patients may not strictly fit into that box. So, for instance, patients might have, if they've travelled in from overseas and they've got a pneumonia, you have to be concerned about the possibility of them having been exposed to antimicrobial resistance in those countries.' P12<br><br>"If they've got like multiple infections at the same time or they tried antibiotics and they've failed, so they need to try something else. Or they've got a culture and they've cultured something strange that's not typical that the guideline therapy covers. Again like, considering the risk of immunosuppression. Multiple infections all at once." P5

			"And occasionally, you might be thinking, well, there could be a second pathology, like a UTI as well as pneumonia, and then you want to pick something that's going to cover both. So I might use ampicillin in that person rather than BenPen [benzylpenicillin] because I'm thinking that's more likely to better cover UTI as well as a chest infection." P16<br><br>"I think the guidelines suggest doxycycline, but I tend to use azithromycin... that's not just for its antimicrobial property, but I think that azithromycin has the best evidence for improving outcomes in community acquired pneumonia, regardless of the actual microbiology... I'm also using it in the knowledge that it's probably actually improving outcomes, because of its immunomodulatory effects." P10<br><br>"And we use prophylactic antibiotics as well such as azithromycin for bronchiectasis patients, both CF and non-CF related, and the evidence shows clearly that long term use prevents the exacerbation in some studies and suggests it can also improve quality of life and to an extent improve the FEV1 (the lung function)." P7<br><br>"So, it's mainly, I guess, if you've tried what's in the guidelines, and that hasn't worked or if you do have like, quite complex patients that don't necessarily fit the nice categories that are set out in the TGs." P11<br><br>"If the patient is deteriorating and your sort of last line of defence is really to give them some antibiotics and hope that they turn the other way. That's probably when it's reasonable. I guess patients might sometimes not clinically fit the picture, but there are instances when you know that's all you've got and you want to just try to make your patients survive." P3
Barriers to optimal prescribing	Diagnostic limitations in RTIs	Participants discussion of limitations with the state of current diagnostics available to guide antimicrobial choices.	"Microbial culture is poorly sensitive and poorly specific, particularly in COPD." P10<br><br>"I don't think there's very good pathology or micro so sometimes one big influencing factor is, if a patient has a virus, you'll always hear the doctor say that there could be superimposed bacterial pneumonia, and it's very hard to...

			There's no real clear-cut markers to differentiate between the two. There's some markers, but they're not necessarily sensitive or specific. So that's a big one - so no good markers. You might not get micro in those instances, so it is hard to determine what exactly you're treating as well." P6<br><br>"And sputum cultures probably have little to no utility in managing acute pneumonia, both because of the way that those samples are handled by the micro lab and the time it takes for the results to come back." P16<br><br>"I think there's a delay in presentation, a delay in recognition, a delay in imaging to help identify the disease." P15<br><br>"Zero capacity [for therapeutic drug monitoring] throughout my LHD [local health district] even at my large metropolitan hospital." P8<br>"I know that much of our micro stuff gets sent to [Western Sydney metropolitan hospital]. And that can be an issue because, for instance, AFBs [acid fast bacillus]... have to get in a car and go to Sydney, and if it's a Friday, they don't get there till the Monday or the Tuesday, and then no one's looked at them... So that is something that can cause delays in, you know, diagnosis or knowing what you're treating." P17<br><br>"So, there is only pathology labs at three hospitals. So, the rest all need to travel places... A group of our most rural hospitals that are outliers and send everything to Sydney to [pathology laboratory]. So, if a blood culture is taken after the plane leaves on a Friday, so for example, if a blood or any culture was done on Friday at 5pm, it doesn't go to Sydney until Monday morning, and then it doesn't get processed for however long that takes. So, it could be a full week before you have any results whatsoever... they're flying blind most of the time just needing to use empirical therapy." P8<br><br>"Because I'm relying, like I say, I'm relying on the eTG people, and they're looking at Australia wide data. Might [my local health district] be different to that? Yeah, I think there's plausible reasons why it might be. Do I think it is? No, not substantially. But, you know, if I could see what the antibiogram that

			the eTG was basing their recommendations on, and the antibiogram that was relevant in my area, that might sway my prescribing?" P11
	Resource and staffing limitations	Participants' discussions of how resourcing within medical and nursing teams impacted antimicrobial decisions.	"So other people see the patients over the weekend. And very often, by the time I've gotten back to the hospital, by the time my registrar calls me on a Monday or I see the patient on a Tuesday, someone, usually more junior than me, has decided to change them to ceftriaxone or oh, my God Tazocin [piperacillin-tazobactam], because they've decided that the patient isn't getting better." P11<br><br>"If you're practicing in a rural sort of general hospital and you've got a complex patient, and you know that it might take like a few hours for them to get to sort of a tertiary centre with specialist resources, I guess you might not want to waste time… that's sort a factor in starting IV therapy. You want to give them something good to start off. You're not going to sort of watch and wait. Like your level of watch and wait to treat is probably a factor in how you can escalate." P5<br><br>"When a patient does start to deteriorate, a phone call would be made to the referral hospital… Because of the bed shortage, they have to hold on to these patients for much, much longer… So, the patients are stuck in these rural sites with less medical oversight for a longer period of time. So again, care is escalated, they're going to have that extra coverage because they don't know how long it's going to be before they can get a bed at the next hospital down the road." P8<br><br>"So, I guess, not having an ID physician on site, the cultures taking ages to come back… Mind you, we used to have a clinical microbiologist in [regional town] in our lab, who would regularly contact our ID physician, but she since resigned a few years ago, and she was never replaced by anyone… But yeah, I guess that's probably one of the main reasons why… our usage is over the average compared other hospitals." P4<br><br>"And then if I have queries, I'll generally run up past the Infectious Diseases. The only issue is he's one person amongst many, many patients. So, he does

			get quite inundated. So that might be a mental barrier to sort of disturbing him with questions." P17<br><br>"But also, because we don't have an ID physician on site who can give them a tap on the shoulder and say, "Hey, the guidelines say this but you are doing this, why is that?" kind of thing. We don't have that, I guess, manpower to kind of enforce that." P4<br><br>"I do use the Hospital in the Home service for people with pneumonia and that that hurts my soul a bit. Because the Hospital in the Home service can only give up to [twice a day] antibiotics intravenously, and keep an eye on people. And there are some people that you're like 'yeah, I think you're well enough, but I'd prefer to give you intravenous antibiotics' because I feel like they work better and the patients feel like they work better. But because Hospital in the Home can only do [twice a day] at a maximum, then my only option for home is ceftriaxone." P11<br><br>"Sometimes people get put on Hospital in the Home, and that means that they're in a way kind of forced on to broader therapy like ceftriaxone because the nurses only can do once a day visits, and so rather than getting BenPen [benzylpenicillin], which would have been adequate, they get given ceftriaxone, which they probably don't need. And it means that they often also then get this predetermined duration of therapy for five days, when if you were seeing them in the hospital each day, you might, the next day, say "well, they look well enough, put them on orals". But that's kind of in the "too hard" basket for HITH to manage." P16
	Healthcare setting	Participants from private and regional hospitals described how their setting impacted antimicrobial prescribing.	"But I think it's just a bit hard when you know you've got surgeons working in the private sector as well as our hospital, and in the private sector they often don't have an AMS Pharmacist or an ID physician at times, and there's no one kind of saying "no, you shouldn't be doing that". But so that's why they carry their practice, you know, from private to public hospitals. So, it's really hard to change that kind of practice." P4

			“Prescribers [in private hospitals] are not employed by the hospital… and therefore, the patients are their own and the risk and responsibility are their own. And so, it makes it very difficult for the hospital to then enforce everyone has to comply.” P9<br><br>“Just because you don't want the risk that a patient might deteriorate and get missed. And often we don't have things like antibiotic stewardship programs in a private hospital. So, there won't be ID physicians looking over your shoulder, sort of questioning your use of antibiotics. Whereas you're likely to get that in a public hospital, where, for example, if you've been using Tazocin [piperacillin-tazobactam] for 10 days, the team's likely going to get a call saying "what's the plan here? Why is he still on antibiotics?" P14<br><br>“So, a lot of the GP VMOs [visiting medical officer] use prescribing software that is purchased by their practice, which does give dose recommendations and therapeutic advice from eMIMS. eMIMS is obviously not up to date, or has dosing advice on medications from 20 years ago. And so, for example, amoxicillin, that's the easiest one. Their doses will always say give 500 milligrams TDS [three times a day]. And how we know in CAP [community-acquired pneumonia] you need a gram.” P8<br><br>“So, GP VMOs [visiting medical officer] are not located at the hospital. They come up when there is a requirement for them to visit hospital. So, they might be there for an hour in the morning and then they will go back to their practice, which most of the time, is off-site. And so those patients are being completely monitored by nursing staff. And there's a lot of reliance on the nursing staff to escalate and phone them if the patient deteriorates. So, because they're not physically there, and there is no medical officer on site, there's a lot of fear that the patient will deteriorate while the medical officer is off-site. So, they escalate care. In their mind, they feel like they're preventing them being called back in for a deteriorating patient.” P8<br><br>“And that's because we've got like locum GP prescribers coming through who aren't… They've got their way of doing things, and they've always had their way

			of doing things, and to be honest, nobody can tell them to change, because… I don't know. The hospital's so hard-staffed, you know. It's so hard for them to get any consultants. They're not going to say "Oh, well, we're not going to renew your contract if you keep prescribing against the guidelines." So, it's a tough environment. Especially with me being remote. It's very easy for people to just go "Eh, don't worry about it. It's just the guy on the end of the phone. What's he going to do?" P2<br><br>"Private, you're more likely to give them IV antibiotics because I think, as opposed to what most people think, private hospitals are not good places for very sick people, because there's less doctors on the wards, there aren't the same sort of ICU support you have in a private hospital compared to a public hospital, so if something does go wrong, it is often picked up later in a private hospital. And so, you tend to create more safe in a private hospital and you'd use more IV antibiotics for longer compared to a public hospital." P14<br><br>"So often the physician may not visit for a couple of days, and then there's nobody to check, so the intravenous antibiotic will just keep going. For example, they start on a Thursday, it might not get changed until Monday, just because the physician hasn't been around on the weekend and nobody has seen the patient on the weekend." P14
	Patient demand	Patient demand for antimicrobial therapy could impact antimicrobial decisions.	"In the private, occasionally the patients will be very demanding. So, they'll demand IV antibiotics. You have to work hard to convince them that they don't need the IV, that the tablets will suffice, or they don't need any antibiotics at all. Where public land, it's a lot easier to do that. That's just the way it works. So that can be challenging sometimes, the demand for IV antibiotics. It doesn't often bother me, I will often stop the antibiotics when I think it's not required." P14<br><br>"One barrier is patient expectations, which I assume you've heard many times before. They want a script for something, and, you know, they expect that from their GP, they expect that from you. And it can be challenging to combat that in many ways. And that's something that's been happening for a long time. People can equate cough and sputum with infection. And it can be quite

			difficult in someone who's smoking to explain to them that it doesn't mean they've got an infection, it means they should stop smoking. So, I suppose that's the only other thing and I think that's probably a universal problem in a lot of ways." P17<br><br>"If patients come into hospital, they'll think they're sick, they'll think they need IV antibiotics, so that might be an influencing factor for why they wanna continue giving it in terms of patient care." P6
	Clinical experience and knowledge	Physicians' clinical experience, knowledge, and autonomy influenced their decisions.	"It depends on the prescriber, I guess, and their awareness of the issue of antimicrobial resistance and the issue of spectrum. And some doctors may just prescribe broader just because they think that that's likely to quickly get on top of the problem. And hit them hard as some of them say. But then they don't always consider the fact that you hit them hard, but then if they don't respond to that, there's no room to escalate, if you go very broad." P12<br><br>"It could be a lack of knowledge around prescribing of antimicrobials. If you're a junior doctor reviewing your patient, it might not be very clear cut when to step down IV [intravenous] to oral." P6<br><br>"I guess there's a sort of perception that benzylpenicillin might not be sufficient for the mild and moderate pneumonias." P3<br><br>"A lot of overseas doctors, whose knowledge may not be as good… and that makes it more difficult as well." P15<br><br>"And some of [the doctors] might be from overseas as well. So maybe they have a different antimicrobial regime that they use overseas, they could have more resistant organisms, and they just thought, like, you know, it's the same here. Which is usually not the case." P1<br><br>"I think within the hospital itself, we often stumble across people who are on what we think is too broad, and we're trying to narrow them down, but sometimes there's a reticence if they're on a pathway, and they probably

			could have gotten away with a narrow spectrum, but they're improving, and maybe you don't then narrow them back down.” P17<br><br>“If in ED [emergency department] they're unwell, the choice of prescribing is actually driven by ED, and then they either are continued on, and no one really reviews them. I think sometimes the doctors don't even realize the patient’s on it because there's no mandated review of medication charts, which is eMeds [electronic medication management software]. So eMeds I think has a part in it.” P6<br><br>“We have some awful respiratory physicians in regard to trying to work together that they feel like we're taking away their autonomy, and so that's just been a long kind of process that we still haven't solved with the ones that are less understanding of what we're trying to do. And that's to help rather than hinder and impede on their autonomy.” P13<br><br>“Respiratory, obviously, are the expert in the lungs. And so, I guess, with any kind of specialists within a particular area in which they're interested, so lungs, they might feel like they know everything about the lungs, including infections. Does that make sense? So I guess they feel like they probably know more about the organ in which the infection is occurring, even though ID may be the experts of general infectious diseases.” P13<br><br>“I guess it's just comes down to the culture and also their personal experiences, I guess. I think they're so used to using ceftriaxone and azithromycin, it works fine. It's hard to get them to change.” P3<br><br>“But where there's resistance to change, this tends to be what is the reasoning behind the resistance to following guidelines.” P9
	Time pressures	Time constraints impacted the ability to perform thorough patient and guideline assessment.	“Yeah, they might not have time to read the guidelines as well. I think if the guidelines are too wordy say, “it's too much, it's too hard”.” P6<br><br>“Freestyle prescribing is a lot of the problem too, because it relies on the clinicians to either have the guidelines in front of them on a different page or

			use their memory. Most of the time, because they're so busy, they're using their memory and their memory is not great for every single respiratory tract infection.” P8
Enablers of optimal prescribing	Relationship between physician and AMS team	How the working relationship between physicians and the AMS team impacted antimicrobial choices.	“In our ED [emergency department] AMS rounds we try to focus and see those CAP [community-acquired pneumonia] patients that come in just to intervene at the point of diagnosis as well, so getting the antibiotic right from the beginning. There's been studies shown as well that demonstrates when antibiotics are started in ED, that they're unlikely to change on the ward, they just get continued through. So, I guess if you get the choice right from the start, they tend to not change it.” P3<br><br>“A pharmacist or an AMS pharmacist or an ID physician at the time of prescribing like that would be ideal, because we often have patients on, you know, 3 or 4 days of ceftriaxone, and then by then they only submit an Ezy [electronic antimicrobial restriction and approval software] then, and then by day 4, it gets rejected. So, you know, patients have gone 3 or 4 days deep on inappropriate antibiotics. So, I think in an ideal world I would love to have someone reviewing the prescribing at the time of prescribing to make sure that the guidelines are strictly followed.” P4<br><br>“Yeah. So, I think this having a conversation, having a dialogue with them, and that's what I'm planning to do next year. Do audits and present the results, and communicate with them, and try to gauge an idea of where they get their evidence from and then work with them. So just a lot of education and some conversations around that.” P1<br><br>“The AMS lead has engaged the head of respiratory department and he’s keen to do something about the antibiotic usage as well. So, I think they're open for conversation, maybe except for one. But most of them at the pretty open to that conversation and discussion. And if we can present evidence and show them that you know, we can use BenPen [benzylpenicillin], and we can step down to orals earlier, I think they should be receptive. But it needs to come from the ID consultant as well, not just me.” P1

			“I would like to see more collaboration between our department, especially CF department, and the Infectious Disease team department, actually communicate together and talk things through and discuss interesting topics. I think that's quite important. I think Infectious Disease team wanted to collaborate, especially as respiratory infections are everyday things. I think it's probably best to meet quarterly or at least twice a year or even more and then discuss some different topics and do a journal club together. Yeah, I think that would be good. More interaction.” P7<br><br>“We [the AMS team] have an excellent relationship now [with the lung transplant team], it's taken years, like almost the entire time I've been here to develop a really good relationship with my lung transplant teams. So, you know, we can always have that discussion. And while we might not always 100% agree with each other, we can still at least see each other's point and usually compromise and make a compromise we're happy with. So, I think the weekly meetings have been really beneficial.” P13<br><br>“I think what I found effective at other places in the past is getting the prescribers involved in what you're doing from the start, Like, if you're doing a project to try and improve something in respiratory prescribing, get them involved from the start. And then you've got this like co-design type strategy. I mean you can put out all these guidelines. But you got to see where they're coming from, too.” P5<br><br>“If they've bought into it, if we're doing some work, for instance in emergency, when we did the community-acquired pneumonia project, we had pharmacy working with Emergency working with respiratory. Everyone was on board. The head of Emergency was very supportive, sort of did a lot of the work in Emergency. Respiratory was involved and they were very much had buy in. And so, we found that project worked well. And it wasn't driven by Pharmacy. So, I think those are the scenarios where it works best.” P9
	Provide feedback on prescribing habits	Provide feedback to physicians on their prescribing habits and	“The other thing that sometimes works is to have anonymised feedback, so that I get my feedback about what I prescribed in the past six months, or 12 months, or whatever, and that is compared to all of my peer colleagues at my

		compare these to others in their team or hospital.	hospital. And so, you know, maybe that would be a way of providing feedback to people that's individualised and anonymised about where their practice sits with reference to everyone else in their peer hospital." P11<br><br>"One thing I would really like to do one day is, in the local cohort, is to just to compare the prescribing patterns between consultants, and then show outcomes between the patients. Because at the end of the day, they usually probably treat the same number of pneumonia patients, and then they all use different therapies, for example, and then show the difference between outcomes. But it's just like a longer-term thing that needs to be looked at and seeing like "did they re-present? Did they actually get C diff? Do they now have resistant Pseudomonas?" kind of things." P13
	Providing evidence of harms of suboptimal prescribing	Provide evidence of harms of inappropriate prescribing on the patient and health system.	"Like them just knowing the potential risks of what might be bad about antibiotics being used inappropriately, I guess you could fast forward 50 years and go like "hey, you used so much Tazocin [piperacillin-tazobactam] that, you know, that's gone as an option" for example. Or, you know, just seeing all the people you caused C diff, because they might not even know that as well, because they've been discharged from hospital. Or the resistance they caused on whatever demographic that they're in, "okay, well, all these patients are now resistant to Tazocin because of this". Things happen when something bad happens, right? So that's why I think it's very much the harms need to be shown of what's going on." P13<br><br>"And so now I'm trying to like give evidence. You know, this practice actually has caused C diff [*Clostridioides difficile*] in this case and giving 72 hours of cefazolin on all knee replacement is actually increasing your length of stay. So, I'm trying to use other angles to try and say "Well, like, you're actually harming people in other ways" rather than just me whinging about antimicrobial resistance." P2
	Increased access to data and diagnostics	How access to antimicrobial data and diagnostics informed antimicrobial decisions.	"You know, like my idea about looking at penicillin resistance in my population? If the lab, and it wouldn't be that hard, published on a regular basis, you know, every six months or something, the amount of antimicrobial resistance noted in, you know, *pneumococcus, Haemophilus,* whatever, in the past six months, and published that, then, you know, people could start to

			make decisions based on evidence, on science, on what's in the local community, rather than on what they reckon, because that's a fair bit of what's happening now. So that might be a sort of scientific guideline-based way to do it.” P11<br><br>“Yeah, I think it would be really nice to have ready access to local data about, you know, what are the most common organisms in that in that season? You know, is there any escalation in resistance? Or, you know, in a way demystify, like, if you think that that is resistant, no, in fact, this will still be responsive to XY or Z.” P17
	Education	Education to physicians and other healthcare professionals on AMS.	“So, a lot of education and re-education. So, I would say, there's a lot of doctors obviously rotating through and whatnot, and sometimes the ATs or the BPTs might not review the antibiotics, or just continue as per consultant. But if we continue to re-educate, maybe they can also flag it. Having a top-down approach as well.” P3<br><br>“And also education, not just for the JMOs. I feel the JMOs, when I do interventions, and I speak with them they're like… if I say “Oh, the guidelines say this and this” and they say “no, I agree with you. I totally agree with you. I agree it's wrong, and that it shouldn't be done. But this is what the boss wants”. So I feel like it should come from the heads of department in terms of like, you know, creating awareness and education.” P4<br><br>“I guess when the last Therapeutic Guidelines came out, and there was very clear instructions for when we were de-escalating in Community Acquired Pneumonia to use the one-gram amoxicillin TDS, and there was education to the junior doctors, the pharmacists, and respiratory, just to remind them that this was in the new guidelines. And we saw a real shift in it. And that was just a lot of education, but also groundwork. So, lots of conversations saying 'oh, we've noticed Augmentin Duo Forte was the step-down. Did you know…?'. Anda couple of those conversations. And now we're seeing appropriate step-down.” P9

			“Also, yeah, I guess, trying to get pharmacists involved as well. So educating pharmacists to intervene and take part in AMS as well.” P3
	Decision support tools	Antimicrobial decision support tools built-in to prescribing software to guide optimal decisions.	“PowerPlan built into eMeds [electronic medication management software] just with the antibiotic guidelines for CAP.” P3<br><br>“I think there needs to be inbuilt algorithms... that kind of guides the prescriber to charting the right thing. So, I think if a prescriber’s charting something, if the program was smart enough to detect oxygen saturations, actually tell the prescriber... I mean overtakes their skill set I suppose... this is likely mild to moderate, you know. Interpret the chest x-ray. You should chart... these are your options. Patient has a pen allergy, patient don't have a pen allergy. And actually have the guidelines in-built into the prescribing software would be the best way to have prescriber adherence. Yeah. And then, if you wanted to, you know, not do that, then you could say no, and then justify your rationale...” P6<br><br>“I want to see health decision support pathways in place of guidelines. I want to see machine learning and direct relational rules to identify patients of interest on your ward list and to have patient identification be a part of workflow rather than interruptive alert messages which are not effective. I want guidelines to be a part of the patient review process.” P2<br><br>“So, if you want to apply for a broad-spectrum drug then you say “I'm treating CAP”, and then it would tell you, “These are your options for CAP”, and you would select. Does your patient have bilateral chest x-ray changes? Does your patient have a pen allergy? Does your patient have you know, oxygen sats less than 90%? And blood pressure? This this. And then it kind of goes down the algorithm of your patient likely has severe pneumonia. You should chart this and this and do these tests. I've seen that before. However, it's separate to PowerChart. So, it does mandate logging into a different program.” P6<br><br>“So having that up with a risk assessment which helps them with diagnosis as well, and then that pre-populating their risk of mortality and then recommending an antibiotic choice would be helpful.” P3

			“Where you might plug in the patient's age, of course, there are many permutations and combinations of immunocompromised.... So, you know, if it was, say, a 30-year-old with no medical comorbidities, and who's got a community acquired probably bacterial pneumonia, how would you, in terms of a decision, to give them standard therapy versus something super broad? I think it might be more helpful for that type of situation.” P12<br><br>“I mean, again, the obvious one, to me is like decision making tools integrated into the eMR [electronic medical record]. So, for example, you know, the patient has been on ceftriaxone, right, when they came to hospital. The decision-making tool that pops up and says 'patient has had ceftriaxone for 48 hours, consider switching to something else'. That type of thing, again, it's not an instruction, but it's a prompt to kind of go 'oh, actually, you know, it has been 48 hours and the patient's temperature has gone down'. It can even prompt the residents to maybe call me and say 'listen, the patient's temperature has gone down so, can we consider changing antibiotics?' and I'm going to go 'yep, that sounds good to me'. So yeah, using that sort of thing for prompting decisions.” P10<br><br>“Mandatory drop down for ceftriaxone and azithromycin - "You've prescribed a severe community acquired pneumonia antibiotic. Can you please confirm this patient has severe community acquired pneumonia?". Or a feedback system - "You've prescribed to the community acquired pneumonia cover. The observation chart does not reflect this. Please confirm that this is appropriate prescribing?".” P15<br><br>“Yeah, there's also, I guess, ways to try and electronically prompt clinicians to follow the guidelines and go in the right direction, and less opportunity for clinicians to just prescribe freestyle.” P6
