# Supplementary material for: Barriers and Enablers to Optimal Antimicrobial Use in Respiratory Tract Infections
Source: Antibiotics (Basel). 2025 Oct 16;14(10):1039. doi: 10.3390/antibiotics14101039 (PMC12562221; doi:10.3390/antibiotics14101039)
Supplement: Supplementary file 1 [file antibiotics-14-01039-s001.zip › Supplement 2 - COREQ Criteria.pdf]

COREQ Criteria	Guide Questions	Response
**Domain 1: Research team and reflexivity**		
Personal characteristics		
1. Interviewer/facilitator	Which author/s conducted the interview or focus group?	• All interviews were conducted by SR.
2. Credentials	What were the researcher's credentials? E.g. PhD, MD	• SR has a Bachelor of Pharmacy with First Class Honours and the University Medal from the University of Sydney.<br>• She is a registered pharmacist.<br>• She is also a member of the Sydney Infectious Diseases Institute.
3. Occupation	What was their occupation at the time of the study?	• SR is a clinical pharmacist in a large teaching hospital and PhD student with the University of Sydney School of Pharmacy.
4. Gender	Was the researcher male or female?	• SR identifies as female.
5. Experience and training	What experience or training did the researcher have?	• SR is a clinical pharmacist with detailed knowledge and experience in the antimicrobial and respiratory fields.<br>• SR undertook qualitative research training through the University of Sydney, read literature on the interview process, and performed pilot interviews with supervision and feedback from her PhD supervisor prior to commencement of interviews.<br>• JGC, JWA, and PA have extensive research experience spanning the fields of pharmacy and medicine, and research methods, including qualitative research.
Relationship with participants		
6. Relationship established	Was a relationship established prior to study commencement?	• SR has worked with one of the AMS pharmacists prior to study commencement. At the time of the interview, they were not working together. The

		relationship was only professional in nature and was not believed to influence the study in any way.<br>• The Participant Information Sheet contained some background information about SR. Before the interview commenced, SR again introduced herself and provided some information on her background, as well as her role and the interviewees role in the study.<br>• SR read through the study objectives and allowed participants the opportunity to ask questions before the interview commenced.
7. Participant knowledge of the interviewer	What did the participants know about the researcher? e.g. personal goals, reasons for doing the research	• The participants received a Participant Information Sheet prior to the interview with a description of the study including aims of the research and background information on the researchers.<br>• This background information included the fact that SR is a PhD student and clinical pharmacist.
8. Interviewer characteristics	What characteristics were reported about the interviewer/facilitator? e.g. Bias, assumptions, reasons and interests in the research topic	• SR is a higher degree research student and clinical pharmacist whose background may have influenced interpretation and analysis of data.<br>• Their expertise and understanding of the antimicrobial prescribing process had the potential to influence interactions with and questioning of participants. However, this was believed to be advantageous to participant interactions and data analysis, rather than hinder it.<br>• SR had qualitative research training prior to commencing interviews and considerations were given to these personal biases and controlling for them.

**Domain 2: Study design**		
Theoretical framework		
9. Methodological orientation and Theory	What methodological orientation was stated to underpin the study? e.g. grounded theory, discourse analysis, ethnography, phenomenology, content analysis	• No specific theories underpinned the study design or interview questions.<br>• After data collection was completed, thematic analysis as described by Braun and Clarke(18) was used to analyse the data, following an inductive qualitative approach.
Participant selection		
10. Sampling	How were participants selected? e.g. purposive, convenience, consecutive, snowball	• Purposive sampling of AMS pharmacists and respiratory and infectious diseases staff specialists followed by snowball sampling was used for recruitment.<br>• This aimed to recruit participants from a broad range of demographic and clinical backgrounds to ensure breadth of the sample.
11. Method of approach	How were participants approached? e.g. face-to-face, telephone, mail, email	• The study was advertised through professional organisations (e.g. Advanced Pharmacy Australia [formerly the Society of Hospital Pharmacists of Australia]),through the researchers' professional networks, and snowballing.<br>• Interested participants were invited to contact SR via email for further information about the study and to organise an interview time.
12. Sample size	How many participants were in the study?	• There were 17 participants in total.
13. Non-participation	How many people refused to participate or dropped out? Reasons?	• No participants who agreed to an interview and read and signed the Consent Form refused or withdrew from the study.
Setting		

14. Setting of data collection	Where was the data collected? e.g. home, clinic, workplace	• All interviews were conducted virtually through Zoom or Microsoft Teams meeting software. Interview participants were either at their workplace or at home during interviews.<br>• SR was either in her office or at home during interviews.
15. Presence of non-participants	Was anyone else present besides the participants and researchers?	• Only SR and the participant were present at each interview.
16. Description of sample	What are the important characteristics of the sample? e.g. demographic data, date	• Demographic information was collected from each participant including their gender, role, workplace setting, and years of experience.<br>• This was collected to ensure a broad sample and to analyse trends and differences in responses based on demographics.<br>• Participant demographics are reported in the first paragraph of the Results.
Data collection		
17. Interview guide	Were questions, prompts, guides provided by the authors? Was it pilot tested?	• Interviews were chosen as the data collection tool to gain a rich understanding of the factors influencing antimicrobial decision making and inappropriate antimicrobial use.<br>• The Participant Information Sheet contained some information about the topics that would be discussed in the interviews, but no specific questions or interview guides were supplied prior to the interviews.<br>• Two semi-structured interview guides were prepared, one for physicians and one for AMS pharmacists.<br>• These were pilot tested with one physician and three pharmacists. Feedback was obtained and minimal adjustments to the wording of questions

		were required. The interview guides are located in Appendix B. Questions specific to this study have been highlighted.
18. Repeat interviews	Were repeat interviews carried out? If yes, how many?	• No repeat interviews were conducted.
19. Audio/visual recording	Did the research use audio or visual recording to collect the data?	• All interviews were conducted virtually through Zoom or Microsoft Teams meeting software. Only audio recording was used.<br>• Audio recordings were uploaded to Otter.ai to transcribe the interviews.
20. Field notes	Were field notes made during and/or after the interview or focus group?	• SR kept notes with ideas and themes generated in the first six interviews.<br>• After these interviews a preliminary coding framework was developed and no further notes were taken.
21. Duration	What was the duration of the interviews or focus group?	• Interviews were on average 39 minutes in duration (range, 27 minutes to 48 minutes).
22. Data saturation	Was data saturation discussed?	• Yes, data saturation was reached after 16 interviews.<br>• One more interview was conducted to confirm data saturation.
23. Transcripts returned	Were transcripts returned to participants for comment and/or correction?	• No, transcripts were not returned to participants for comment or correction.
**Domain 3: Analysis and findings**		
Data analysis		
24. Number of data coders	How many data coders coded the data?	• There was a thorough and systematic approach to coding and theme identification with inter-coder reliability testing and peer debriefing.<br>• SR coded all 17 transcripts. To confirm accuracy of the coding process, PA coded two transcripts and a coding comparison was undertaken. Each code from the two transcripts were discussed in

		detail by SR and PA to ensure similar data was extracted from the transcripts.<br>• Codes were sufficiently similar in the two transcripts and no further coding comparison was necessary.
25. Description of the coding tree	Did authors provide a description of the coding tree?	• Yes, Table 1 shows the overall themes and sub-themes and a list of representative quotes for each.
26. Derivation of themes	Were themes identified in advance or derived from the data?	• Themes were derived iteratively from the data.<br>• First, interesting and relevant sections of transcripts were coded in granular detail. Codes were then reviewed and related codes were linked together to begin formation of themes. These relationships were reviewed within the context of the entire dataset and finalised themes were established.<br>• Initially, six transcripts were analysed using this technique to develop a coding framework.
27. Software	What software, if applicable, was used to manage the data?	• NVivo 13 software was used to store transcripts, participant demographics, code and analyse data.
28. Participant checking	Did participants provide feedback on the findings?	• No.
Reporting		
29. Quotations presented	Were participant quotations presented to illustrate the themes / findings? Was each quotation identified? e.g. participant number	• Yes, relevant quotes for each subtheme are incorporated into the text of the Results. The participant number and their role have also been included.<br>• Appendix C provides a more detailed list of all themes and subthemes from the with more supportive quotes.

30. Data and findings consistent	Was there consistency between the data presented and the findings?	• The quotations from the data presented in manuscript were carefully chosen to illustrate the themes and findings of the research.<br>• We believe that there is consistency between the data presented and the findings of the research
31. Clarity of major themes	Were major themes clearly presented in the findings?	• Yes, each major theme is discussed in detail with clear headings and subheadings in the Results.<br>• The breakdown of each theme and subtheme is also made clear in Table 1.
32. Clarity of minor themes	Is there a description of diverse cases or discussion of minor themes?	• Where there were diverse cases, this was highlighted in the manuscript by stating that only participants from certain settings expressed this opinion.
