# Supplementary material for: Barriers and Enablers to Optimal Antimicrobial Use in Respiratory Tract Infections
Source: Antibiotics (Basel). 2025 Oct 16;14(10):1039. doi: 10.3390/antibiotics14101039 (PMC12562221; doi:10.3390/antibiotics14101039)
Supplement: Supplementary file 1 [file antibiotics-14-01039-s001.zip › Supplement 3 - Interview Guides.pdf]

Semi-Structured Interview Guide - Pharmacists

Barriers and Enablers to Optimal Antimicrobial Use in Respiratory Tract Infections

Objectives

1. To identify factors that influence prescribers' decision when prescribing antimicrobials
2. To investigate knowledge of, and opinions about, general recommendations and evidence-based guidelines for prescribing antimicrobials for respiratory tract infections*
3. To identify perceived level of adherence to prescribing guidelines*
4. To identify perceived enablers and barriers to adherence to prescribing guidelines*

Interview Topic	Question(s)
Demographic information (Objective 1)	• What is your primary place of practice?<br>o Do you also work at other sites? Overseas / Rural / Regional<br><br>• How many years have you been practising as a pharmacist?<br>o AMS pharmacist<br><br>• Where did you undertake your pharmacy training?<br>o University / Internship<br><br>• Describe your role within the broader AMS service at your hospital.<br><br>• What is your role in the process of management of patients with respiratory tract infections?
Factors influencing antimicrobial prescribing decisions (Objective 1)	• In a patient diagnosed with a respiratory tract infection, how often do you think you see an antimicrobial prescribed?<br>o Bacterial vs. Viral<br>o Bronchitis / IE asthma or COPD / CAP / Influenza / COVID<br><br>• In a patient diagnosed with a respiratory tract infection, can you please walk me through step-by-step your process of reviewing that patient and their antimicrobial therapy, including all the factors you consider?<br>o Patient factors / System factors / Guidelines / Pathology / Observations / Diagnostic tests?<br>o Choice of antimicrobial / Dose / Duration?
Views on prescribing guidelines (Objective 2)*	• What guidelines or resources do you use to support antimicrobial decisions in respiratory tract infections?<br>o Guidelines / AMS rounds or interventions / Smartphone apps, etc.<br>o Why do you choose to use these guidelines?<br><br>• And what are your thoughts on those recommendations? Do you agree with them generally?
Level of adherence to prescribing guidelines (Objective 3)*	• How often would you say doctors follow the evidence-based uidelines when prescribing antimicrobials for respiratory tract infections?<br><br>• In what situations do you think guidelines are not followed?
Enablers and barriers to adherence to prescribing guidelines (Objective 4)*	• What do you think we could do to improve adherence to prescribing guidelines or help antimicrobials be prescribed more optimally?<br>o Enablers and barriers to guideline adherence and optimal prescribing
Concluding remarks	• Is there anything else you would like to share about decision making around antimicrobials for respiratory tract infections or improving prescribing practices?

*Questions specific to current study.

Semi-Structured Interview Guide - Physician

Barriers and Enablers to Optimal Antimicrobial Use in Respiratory Tract Infections

Objectives

1. To identify factors that influence prescribers' decision when prescribing antimicrobials
2. To investigate knowledge of, and opinions about, general recommendations and evidence-based guidelines for prescribing antimicrobials for respiratory tract infections*
3. To identify perceived level of adherence to prescribing guidelines*
4. To identify perceived enablers and barriers to adherence to prescribing guidelines*

Interview Topic	Question(s)
Demographic information (Objective 1)	• What is your current role?<br>o Speciality<br>• What is your primary place of practice?<br>o Do you also work at other sites? Overseas / Rural / Regioanl<br>• How many years have you been practising as a medical professional?<br>• Where did you undertake your medical training?<br>o University / JMO<br>• What is your role in the process of management of patients with respiratory tract infections?
Factors influencing antimicrobial prescribing decisions (Objective 1)	• In a patient with respiratory tract symptoms, how often do you think you would prescribe them antimicrobials?<br>o Bacterial vs. Viral<br>o Bronchitis / IE asthma or COPD / CAP / Influenza / COVID<br>• In a patient diagnosed with a respiratory tract infection, how you make a decision about whether to prescribe an antimicrobial or not, including all the factors you consider?<br>• When you have decided to prescribe an antimicrobial, can you walk me through step-by-step how you decide what antimicrobial to prescribe, the route, dose, and for what duration, including all the individual factors you consider during that process?<br>o Patient factors / System factors / Guidelines / Pathology / Observations / Diagnostic tests?
Views on prescribing guidelines (Objective 2)*	• What guidelines do you use to support your antimicrobial prescribing decisions in respiratory tract infections?<br>o Guidelines / AMS rounds or interventions / Smartphone apps, etc.<br>o Why do you choose to use these guidelines?<br>• And what are your thoughts on those recommendations? Do you agree with them generally? Thoughts on usability and applicability to your patients?
Level of adherence to prescribing guidelines (Objective 3)*	• How often would you say you follow the evidence-based guidelines when prescribing antimicrobials for respiratory tract infections?<br>• In what situations do you choose not to follow the guidelines?
Enablers and barriers to adherence to prescribing guidelines (Objective 4)*	• What do you think we could do to improve adherence to prescribing guidelines or help antimicrobials be prescribed more optimally?<br>o Enablers and barriers to guideline adherence and optimal prescribing
Concluding remarks	• Is there anything else you would like to share about decision making when prescribing antimicrobials for respiratory tract infections?

*Questions specific to current study.
