# Supplementary material for: Barriers and Enablers to Optimal Antimicrobial Use in Respiratory Tract Infections
Source: Antibiotics (Basel). 2025 Oct 16;14(10):1039. doi: 10.3390/antibiotics14101039 (PMC12562221; doi:10.3390/antibiotics14101039)
Supplement: Supplementary file 1 [file antibiotics-14-01039-s001.zip › Supplement 4 - Barrier and enabler comparison.pdf]

Comparative table of enablers and barriers for AMS pharmacists, and physicians.

Theme	Antimicrobial Stewardship Pharmacist	Physicians
**Enablers**		
Decision support tools		
Increased data		*
Feedback		*
Relationship with AMS team		*
**Barriers**		
Diagnostics		
Resourcing		
Patient demand		*
Knowledge and experience		

*Theme only mentioned by respiratory staff specialists
